# Tissue-Resident Exhausted Effector Memory CD8^+^ T Cells Accumulate in the Retina during Chronic Experimental Autoimmune Uveoretinitis

**DOI:** 10.4049/jimmunol.1301390

**Published:** 2014-04-16

**Authors:** Joanne Boldison, Colin J. Chu, David A. Copland, Philippa J. P. Lait, Tarnjit K. Khera, Andrew D. Dick, Lindsay B. Nicholson

**Affiliations:** * School of Cellular and Molecular Medicine, University of Bristol, Bristol BS8 1TD, United Kingdom;; † Department of Genetics, University College London Institute of Ophthalmology, London EC1V 9EL, United Kingdom; and; ‡ Academic Unit of Ophthalmology, School of Clinical Sciences, University of Bristol, Bristol BS8 1TD, United Kingdom

## Abstract

Experimental autoimmune uveoretinitis is a model for noninfectious posterior segment intraocular inflammation in humans. Although this disease is CD4^+^ T cell dependent, in the persistent phase of disease CD8^+^ T cells accumulate. We show that these are effector memory CD8^+^ T cells that differ from their splenic counterparts with respect to surface expression of CD69, CD103, and Ly6C. These retinal effector memory CD8^+^ T cells have limited cytotoxic effector function, are impaired in their ability to proliferate in response to Ag-specific stimulation, and upregulate programmed death 1 receptor. Treatment with fingolimod (FTY720) during the late phase of disease revealed that retinal CD8^+^ T cells were tissue resident. Despite signs of exhaustion, these cells were functional, as their depletion resulted in an expansion of retinal CD4^+^ T cells and CD11b^+^ macrophages. These results demonstrate that, during chronic autoimmune inflammation, exhausted CD8^+^ T cells become established in the local tissue. They are phenotypically distinct from peripheral CD8^+^ T cells and provide local signals within the tissue by expression of inhibitory receptors such as programmed death 1 that limit persistent inflammation.

## Introduction

Experimental autoimmune uveoretinitis (EAU) is an Ag-specific CD4^+^ T cell–dependent model of noninfectious intraocular inflammation, paralleling clinicopathological features of human uveitis. Animal models have proven useful in probing cellular mechanisms of disease and as a preclinical model for future treatments of human uveitis (1). EAU can be elicited in rodents by immunization with retinal autoantigens, such as retinol-binding protein (RBP)-3, previously known as interphotoreceptor retinoid-binding protein and S-Ag (also known as arrestin). In the C57BL/6 (H-2^b^) mouse model, immunization with the 1–20 RBP-3 peptide and adjuvants provokes persistent disease principally involving the posterior segment of the eye (2).

In murine models of EAU, it is possible to distinguish three phases of disease, the subclinical prodrome, a primary peak, and a period of secondary regulation (3). Secondary regulation is characterized by longstanding changes in the character of immunosurveillance as assessed by the level of immune cell infiltration. It manifests aspects of chronically disordered retinal regeneration, features that are also commonly found in human disease, specifically the development of intraretinal neovascular membranes (4).

Clinical disease in EAU depends on both CD4^+^ T cells and macrophages; depleting either during the prodromal phase prevents progression (5, 6). However, other immune cells also play an important role in regulating disease, including CD8^+^ T cells (7–9). Recently, transcriptional profiling of CD8^+^ T cells from patients with severe autoimmune disease revealed them as a potential biomarker for patients with poor prognosis (10, 11). In EAU and other models of organ-specific autoimmune disease, in which CD8^+^ T cells have been studied, they have been ascribed a variety of roles (12–16). CD8^+^ T cells have been reported to accumulate in late uveitis in rat models of disease, but depletion of these cells from the time of disease induction had little effect, and it remains unclear as to whether the cells regulate or contribute to the persistence of disease (17–19). Recently, there has been a growing awareness of heterogeneity among CD8^+^ T cells that are expanded as part of an acute immune response. The responding population is comprised of a mixture of different subsets that can be classified using cell surface markers, of which effector memory CD8^+^ T cells (T_EM_) cells are the predominant subset that enters peripheral tissues (20, 21). It has been of recent interest to determine the conditions that dictate whether T_EM_ are retained in the target tissue or recirculate from the blood and continually repopulate the peripheral tissues. One outcome of acute viral infection is the generation of a subset of tissue-resident effector memory CD8^+^ T cells (T_RM_) that populate normal and immune privileged peripheral organs such as the gut and the brain following the resolution of infection (22–25). Further studies have revealed subsets of T_RM_ residing in the skin, lung, and salivary glands (26–29). This distinct population of cells has not only been identified in mouse models of infection but also in human mucosal tissue, and, importantly, expression patterns of key markers such as CD103 and CD69 are consistent in humans with those demonstrated in murine models (30, 31). These T_RM_ have been shown to provide protection against infection within the local tissue and limit secondary infection (27, 32). This form of immunological memory has mainly been studied in viral models such as lymphocytic choriomeningitis virus (LCMV) or HSV infection and has not yet been characterized in autoimmune models. With this in mind, we set out to analyze the resident CD8^+^ T cells in the tissue of an autoimmune model, during the persistent phase of disease, in which secondary regulation may be in effect, and assess their role by depleting them from the tissue.

## Materials and Methods

### Mice

C57BL/6J mice were originally obtained from Harlan UK Limited (Oxford, U.K.), and breeding colonies were established within the Animal Services Unit at Bristol University (Bristol, U.K.). Mice were housed in specific pathogen-free conditions with continuously available water and food. Female mice immunized for disease induction were aged between 6 and 8 wk. All mice were kept in the animal house facilities of the University of Bristol, according to the Home Office Regulations. Treatment of animals conformed to United Kingdom legislation and to the Association for Research in Vision and Ophthalmology statement for the Use of Animals in Ophthalmic and Vision Research.

### Reagents

Human RBP-3_1–20_ peptide (GPTHLFQPSLVLDMAKVLLD) and chicken OVA_323–339_ (ISQAVHAAHAEINEAGR) were synthesized by Sigma-Aldrich (Poole, U.K.). Peptide purity was >95% as determined by HPLC. Fingolimod and control analog AAL149 were supplied by Novartis (Basel, Switzerland). Complete medium consisted of RPMI 1640 media supplemented with 10% heat-inactivated FCS, 100 U/ml penicillin-streptomycin, 2 mmol/L L-glutamine, and 5 × 10^−5^ mol/L 2-ME (Invitrogen, Paisley, U.K.).

### EAU induction

C57BL/6J mice were immunized s.c. in one flank with 500 μg 1–20 RBP-3 peptide in water (2% DMSO) emulsified in CFA (1 mg/ml; 1:1 v/v) supplemented with 1.5 mg/ml *Mycobacterium tuberculosis* complete H37 Ra (BD Biosciences, Oxford, U.K.), and 1.5 μg *Bordella pertussis* toxin (Tocris, Bristol, U.K.) was given i.p.

### Topical endoscopic fundal imaging

Photographs were taken of the retina using a method developed previously (33). A 5-cm–long tele-otoscope (1218AA; Karl Storz, Tuttlington, Germany) was attached to a digital camera (Nikon, Tokyo, Japan). A xenon lamp (201315-20; Karl Storz) connected through a flexible optic fiber to the endoscope was used as a light source. The pupils of the mice were dilated using 1% topical tropicamide and 2.5% phenylephrine (Chauvin Pharmaceuticals, Romford, U.K.), and then 0.4% oxybuprocaine (Chauvin Pharmaceuticals) and viscotears were applied (Novartis Pharmaceuticals). The camera was fixed to a bench, and the mouse was positioned at the end of the endoscope and orientated to the correct angle. All pictures were analyzed and cropped using Photoshop CS software (Adobe, Mountain View, CA). Fundal images were scored for inflammatory changes of the optic disc, retinal vessels, retinal lesions, and structural damage; all scores were added together to make a final disease score. This clinical grading system was adapted [from (34)] and has been previously published (35). Scoring was carried out independently by two trained assessors masked to the origin of the data.

### Cell preparations

Spleens were disrupted mechanically through a 40-μm cell strainer, and RBCs were lysed. Eyes were enucleated and carefully cleaned to remove all extraneous connective and vascular tissue. The retinas (including the ciliary body) were microscopically dissected in HBSS media supplemented with 5% FCS. Retinas were then cut into small pieces and digested with 1 ml media containing 0.5 mg/ml collagenase D (Roche, Welwyn Garden City, U.K.) and 750 U/ml DNase I (Sigma-Aldrich) for 20 min at 37°C. Retinal tissue was homogenized and forced through a 70-μm cell strainer with a syringe plunger, to obtain a single-cell suspension, and stained for flow cytometry analysis. Quantification of the number of cells per eye has been described previously (3).

### CFSE proliferation assay

Retinal and splenocyte cell suspensions were prepared, as described above; seeded in 96-well round-bottom plates; and stimulated with either 10 μg/ml RBP-3_1–20_ or OVA_323–339_ in complete medium. Retinal cell preparations (CD45.1) were stained with 0.5 μM CFSE (Invitrogen) and cultured with 2.5 × 10^5^ irradiated APCs from CD45 congenic mice (CD45.2). The congenic markers were used to distinguish the two cell populations. Splenocytes were stained with 0.5 μM CFSE and seeded at 0.5 × 10^6^ per well. Cells were cultured for 4 d at 37°C and with 5% CO_2_ in a humidified atmosphere before flow cytometry staining.

### Fluorescent immunohistochemistry

Eyes were snap frozen in optimal cutting temperature medium and sectioned at 16 μm thickness. Following fixation with 1% paraformaldehyde and nonspecific goat serum block, slides were stained with monoclonal rat anti-mouse CD8α (clone 53-6.7; BD Biosciences). Secondary labeling was performed with an AlexaFluor 546-conjugated goat anti-rat Ab and then costained with DAPI and imaged by either fluorescent (Axio Observer.Z1; Carl Zeiss Microimaging, Jena, Germany) or confocal microscopy (Leica DM5500Q; Leica Microsystems). Images were exported and processed in Adobe Photoshop CS5.5 (Adobe Systems, San Jose, CA).

### Flow cytometry

Cell suspensions were incubated with 24G2 cell supernatant (Fc block) for 10 min at 4°C. Cells were then stained with fluorochrome-conjugated mAb against cell surface markers for 20 min at 4°C. Multiparameter flow cytometry was carried out using different fluorochromes all purchased from BD Biosciences, except for CD107a (gift of D. Morgan, University of Bristol). Granzyme B and programmed death 1 (PD-1) were purchased from eBioscience (Hatfield, U.K.). Cells were resuspended in 7-aminoactinomycin D (7AAD), and dead cells were excluded from analysis by gating on 7AAD^−^ cells. TNF-α, granzyme B, and CD107a were detected after 4 h of activation with PMA (20 ng/ml) and ionomycin (1 μM) with 1 μl/ml Golgiplug (BD Biosciences). IFN-γ production was detected either after PMA/ionomycin stimulation as above or from cells cultured with 10 μg/ml RBP-3_1–20_ or OVA_323–339_ peptides, and with 100 U/ml human rIL-2 for 30 min before the addition of 1 μl/ml Golgiplug for an additional 3 h. Cells were incubated with supernatant 24G2 for 10 min and then stained for cell surface markers for 20 min at 4°C. The cells were then permeabilized with Perm/Fix kit (BD Biosciences), according to the manufacturer’s instructions, and subsequently stained for mAb against intracellular cytokines or appropriate isotype controls. Cell suspensions were then washed and acquired on a flow cytometer (LSR II, FACSDiva software; BD Cytometry Systems). All analyses were performed using FlowJo software (Tree Star, Ashland, OR). The numbers of cells were calculated by reference to known standard curve (3).

### FTY720 treatment

Immunized C57BL/6J mice were treated with 0.3 mg/kg FTY720 or control analog AAL149 in a maximum volume of 150 μl in PBS, by oral gavage. A dose previously titrated to reduce cell infiltrate by 75% (35).

### In vivo CD8 depletion

mAbs were purified from supernatant by growing hybridomas YTS 169.4 and YTS 156.7 (a gift of A. Gallimore, Cardiff University) in complete media with 2.5% FCS. Supernatant was purified using G protein column (GE Healthcare) on AKTAprime chromatography system (GE Healthcare), and concentration of protein was determined by NanoDrop (Thermoscientific). Protein was dialyzed into PBS before injection. Immunized mice were given 250 μg of either both depleting Abs or IgG isotype control by i.p. injection.

### Statistical analysis

All data were analyzed using a Mann–Whitney *U* test, except for retinal CFSE and IFN-γ assays. Data were analyzed using Wilcoxon signed rank test and considered significant when results had a *p* value ≤ 0.05. All graphs and statistical tests were performed using GraphPad Prism (GraphPad Software).

## Results

### Analysis of EAU reveals selective accumulation of CD8^+^ T cells

**FIGURE 1.**
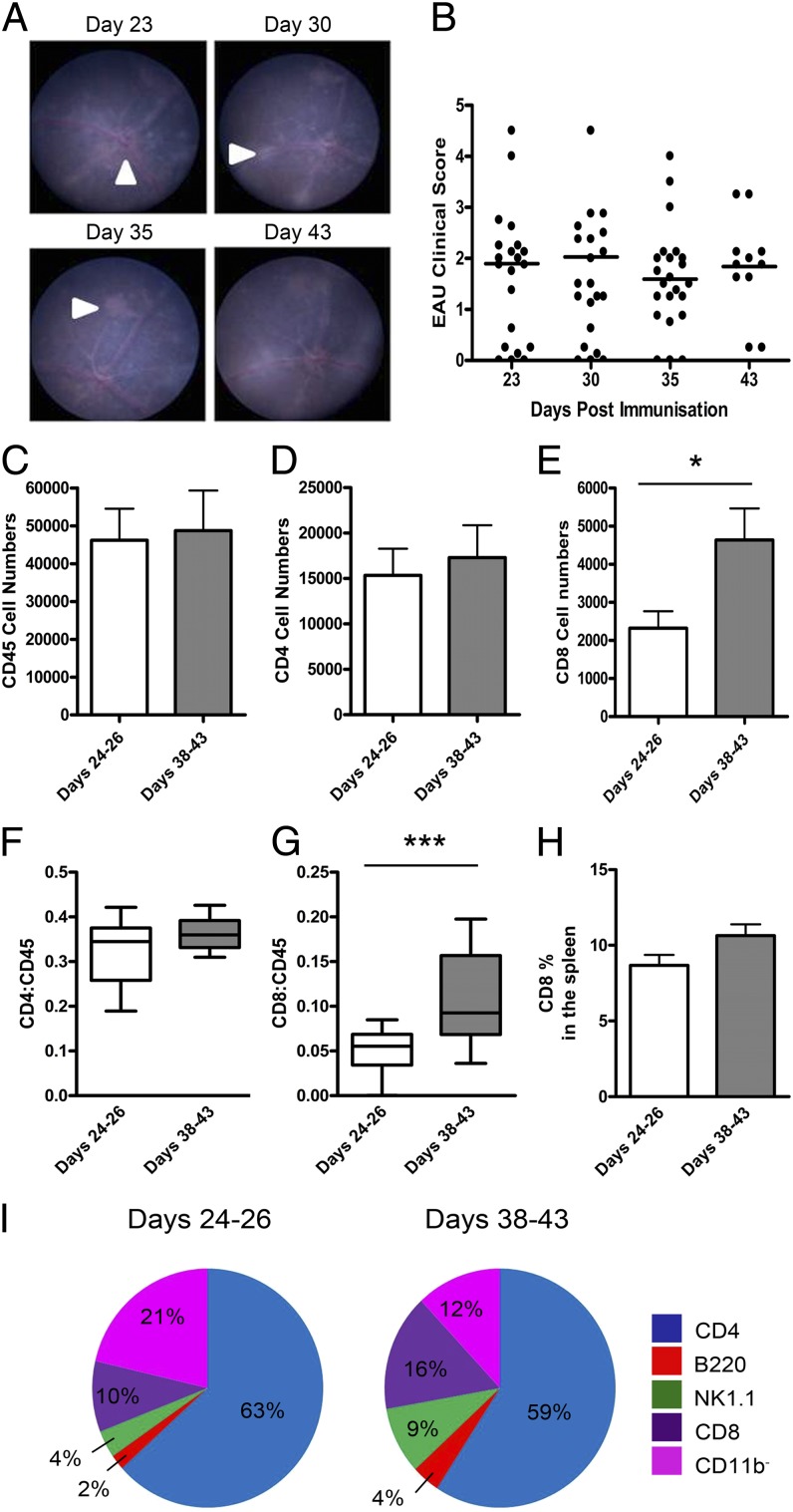
CD8^+^ T cells accumulate in the late phase of EAU. Mice were immunized to induce EAU, after which disease progression was monitored by TEFI and retinal infiltrate characterized by flow cytometry on different days postimmunization. (**A**) Representative TEFI images. Mice display classic EAU features indicated by white arrowheads, including inflamed optic disc (day 23 image), vasculitis (day 30 image), and choroidal lesions (day 35 image). The optic disc has a diameter of ~220 μm. (**B**) EAU disease scores for retinas from different days postimmunization. Black line represents the mean score. (**C**–**E**) Average cell number per retina pooled from different days during EAU for CD45^+^ (C), CD4^+^ (D), and CD8^+^ (E) populations. Data expressed as mean ± SEM. Data are the combination of three independent experiments. Each time point includes a minimum of 23 retinas. (**F** and **G**) Ratios of cell numbers per retina at different time points calculated for CD4:CD45 (F) and CD8:CD45 (G). Box and whisker plots show median, 25th and 75th percentile, and range. (**H**) Percentage of CD8^+^CD3^+^ T cells in the spleen. (**I**) Pie charts represent the average percentages of various retinal cell subpopulations (CD4, B220, NK1.1, and CD8) within the CD11b^−^ parent population. CD11b^−^ populations (pink) were negative for all markers used. All cells were gated on 7AAD^−^CD45^+^ populations. *p < 0.05, ***p < 0.001.

C57BL/6J mice were immunized s.c. with 500 μg RBP-3_1–20_ peptide emulsified in CFA and pertussis toxin. Disease progression was monitored by topical endoscopic fundal imaging (TEFI) throughout the course of the experiment. Representative fundal images display features of classical EAU, including inflammation of the optic disc, vasculitis, and choroidal lesions (Fig. 1A). Retinal inflammation, evaluated by clinical assessment, reached its maximum at day 23 and then remained at a similar level throughout the course of the experiment, which was terminated at day 43 (Fig. 1B). This demonstrates the persistent nature of disease, as previously described (4).

To characterize the underlying disease process in more mechanistic detail, we carried out extensive phenotyping of the cellular infiltrate. We quantified the leukocyte content of the retinas of animals with EAU at two time points, during the primary peak (days 24–26) and the persistent phase (days 36–43), with methodology established in the B10.RIII model (3) (Fig. 1C). Total average CD4^+^ T cell numbers were not different at these two time points (Fig. 1D), whereas CD8^+^ T cell numbers did increase significantly (Fig. 1E) (*p* < 0.05). This accumulation was specific to the inflamed retina, as there was no difference in the percentage of CD8^+^ T cells in the spleen at these two time points (Fig. 1H). Normalizing the CD8^+^ T cells as a fraction of the total CD45^+^ leukocyte population confirmed the selective accumulation of CD8^+^ T cells in the retina (Fig. 1G) compared with CD4^+^ T cells, which showed no selective accumulation (Fig. 1F). The CD8^+^ T cells increased from 10 to 16% of the total CD45^+^CD11b^−^ cell population (Fig. 1I). We concluded that, in the C57BL/6J mouse, persistent uveitis was accompanied by a selective increase of CD8^+^ T cells localized within the retina, the function of which is poorly understood.

### CD8^+^ T cells are localized throughout the retina

**FIGURE 2.**
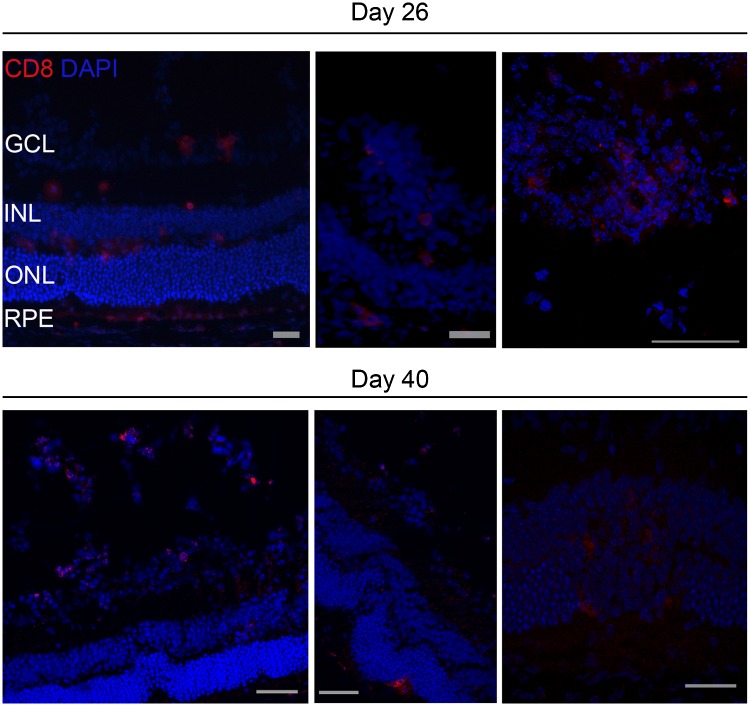
CD8^+^ T cells are localized throughout the retina. Representative images of immunohistochemistry performed on retinal sections from mice (*n* = 7) with EAU at day 26 and day 40 postimmunization show CD8^+^ staining (red) with DAPI counterstain (blue). Staining is prominent around regions of vasculitis, marked intraretinal infiltration, and structural damage. Scale bar, 50 μm.

To determine whether the CD8^+^ T cells accumulate in a specific location or are dispersed throughout the retina, fluorescent immunohistochemistry was performed on retinal sections from mice immunized for EAU (Fig. 2). Confocal images demonstrate the presence of CD8^+^ cells at days 26 and 40 EAU throughout the retinal layers. There were no striking differences in the pattern of accumulation at these two time points. Sections show staining that appeared within regions of vasculitis, retinal folds, and the vitreous. The histology also confirms that the architecture of the retina is relatively well preserved compared with some other models of uveitis (36), providing an ongoing source of retinal Ags to activate specific infiltrating T cells.

### Retinal CD8^+^ T cells show Ag-specific activation

**FIGURE 3.**
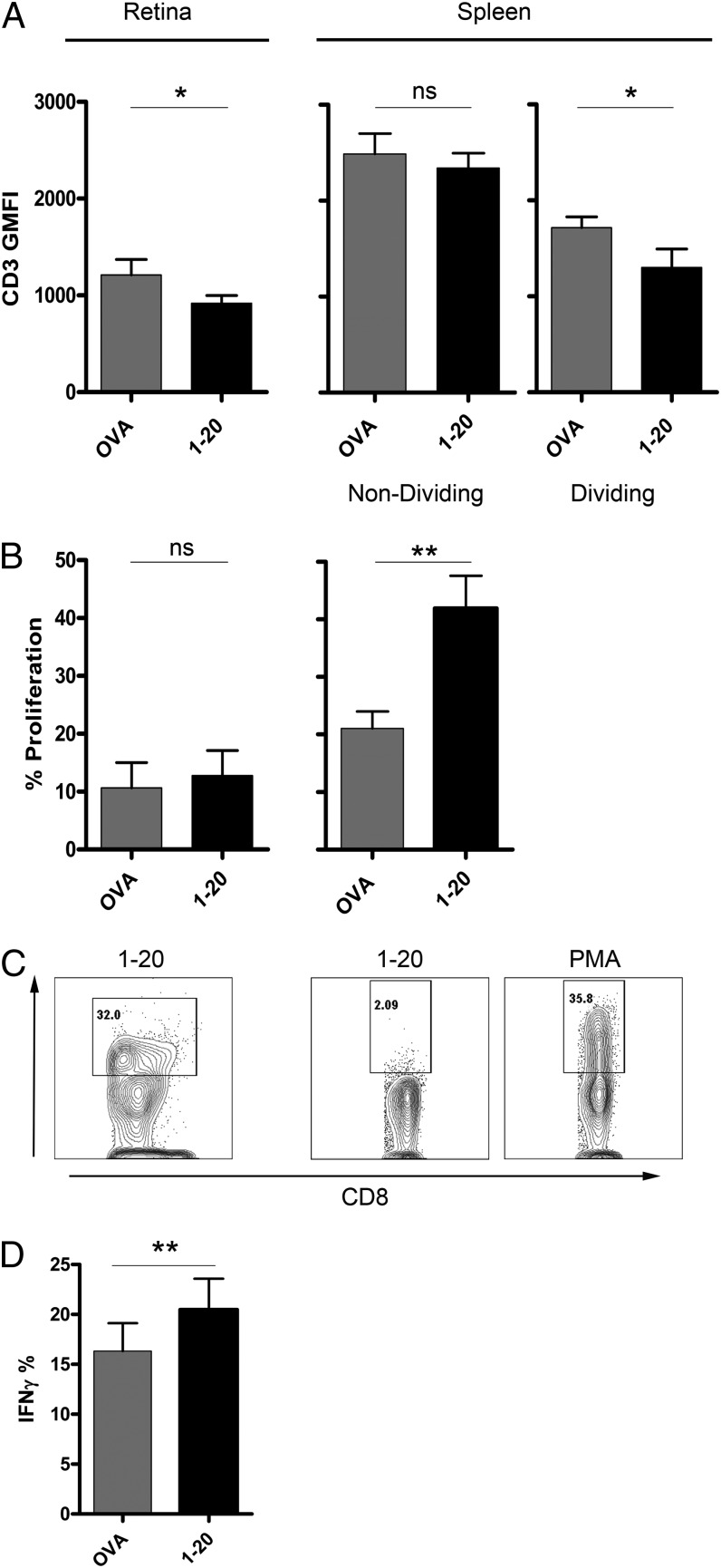
Retinal CD8^+^ T cells show Ag-specific activation. Mice were immunized for EAU, and, at day 40 postimmunization, retinas and spleen were taken to measure proliferation by CFSE dilution (**A** and **B**) or IFN-γ intracellular cytokine staining (**C** and **D**) following stimulation with RBP-3_1–20_ peptide or OVA peptide. All retinal cells were cultured with allelically marked irradiated APCs, which were subsequently gated out from the analysis. Splenocytes stimulated with PMA/ionomycin were used as a positive control. (A) Geometric mean fluorescent intensity (GMFI) for CD3 receptor (B) shows percentage proliferation of CD8^+^ T cells. (C) Representative flow cytometry plots of IFN-γ production from CD8^+^ T cells. (D) Average percentage of IFN-γ–producing CD8^+^ T cells from the retina. All cells gated on live CD8^+^CD3^+^ populations. Data represent three independent experiments, with at least two mice in each experiment. Data expressed as mean ± SEM. *p < 0.05, **p < 0.01. ns, not significant.

Next, we investigated the response of the CD8^+^ T cells to activation with the immunizing peptide. First, we used CFSE staining with Ag-specific stimulation to assess activation and proliferation. Not surprisingly, we detected Ag-specific CD8^+^ T cells in the spleen (37). Segregating splenic CD8^+^ cells into a dividing and nondividing population on the basis of CFSE dilution allowed quantification of CD3 downregulation as a consequence of TCR engagement by cognate peptide (Fig. 3A) (38). Similarly, retinal CD8^+^ T cells displayed a significant downregulation of CD3 when stimulated with 1–20 peptide (Fig. 3A), albeit no proliferation was observed, as we have previously noted with retinal CD4^+^ T cells (39) (Fig. 3B). To test the functional response of these cells, we analyzed retinal Ag-induced IFN-γ intracellular cytokine expression in the presence of IL-2, a method previously used to identify Ag-specific T cells (40). IFN-γ was produced in response to Ag by a much higher percentage of retinal CD8^+^ T cells than their splenic counterparts, achieving levels seen in the spleen only following PMA/ionomycin stimulation (Fig. 3C). Activation of retinal T cells with IL-2 also induced IFN-γ in the presence of nonspecific peptide stimulation, but stimulation with RBP-3_1–20_ elicited significantly more IFN-γ than seen in the control (*p* < 0.01) (Fig. 3D). We also noted that the IFN-γ production from cells obtained from the retina segregates to the CD8 low population, consistent with coreceptor tuning of the cells in response to Ag engagement (41). Taken together, these results indicate that a significant population of retinal CD8^+^ T cells has undergone recent activation.

### Phenotype of retinal T_EM_

**FIGURE 4.**
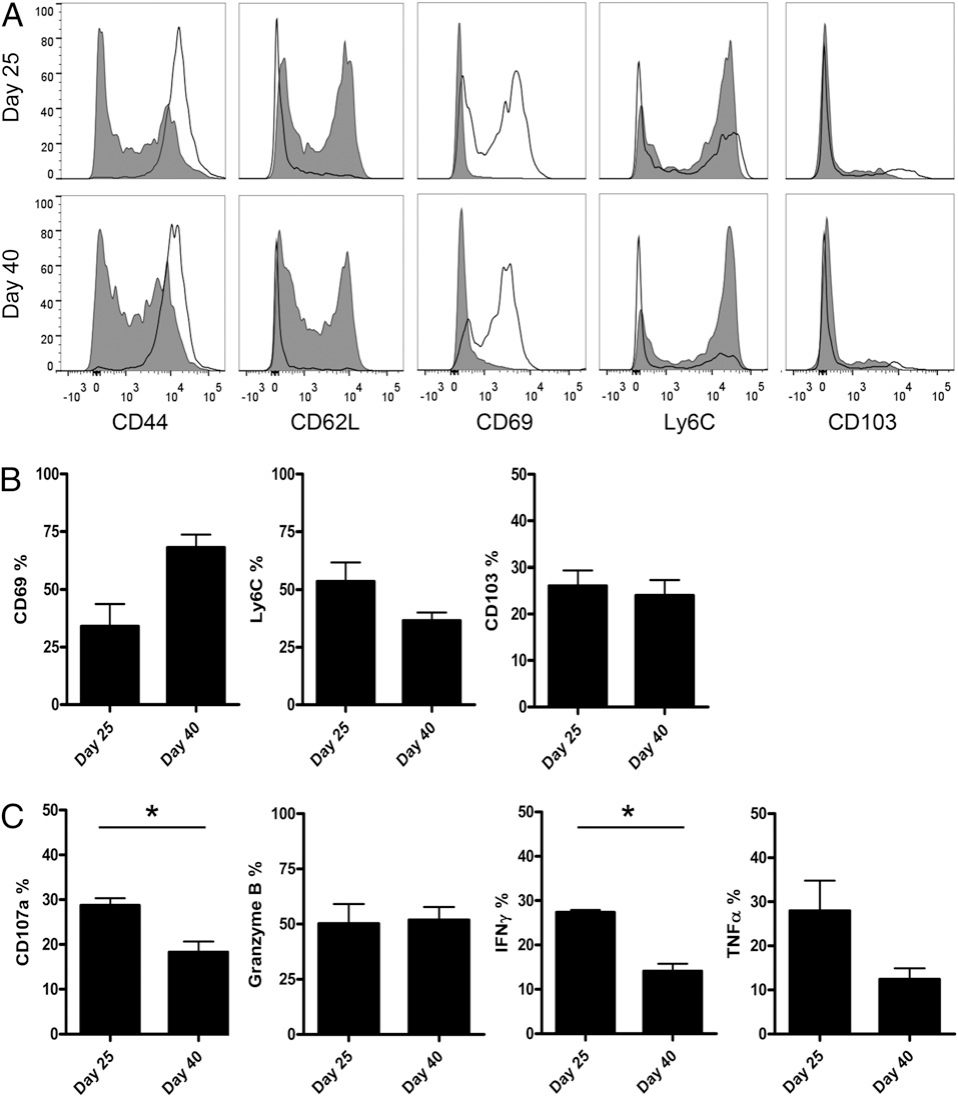
Phenotype of retinal CD8^+^ T cells. Mice were immunized for EAU, and, at different time points, CD8^+^ T cells were examined for the indicated markers. After dissection, retinas were pooled together before analysis, at least three retinas per experiment. (**A**) Representative flow cytometry plots of CD8^+^ T cells isolated from the spleen (gray filled histograms) and the retina (black histograms). CD44 and CD62L markers were gated on CD8^+^CD3^+^ cells; CD69, Ly6C, and CD103 markers were gated on CD8^+^CD3^+^CD44^high^CD62L^low^ populations. (**B** and **C**) Average cell percentage for CD8^+^ T cells prepared from the retina for surface markers (B) and cytotoxic markers and cytokine expression (C). Data represent at least three independent experiments at each time point. Data expressed as mean ± SEM. *p < 0.05.

T_RM_ have a distinct phenotype based on cell surface markers that has been used to characterize their presence in a wide range of different tissues; we used this approach to determine the phenotype of CD8^+^ T cells in the retina. First, we assessed the expression of the well-known memory and activation markers CD44 and CD62L (20) (Fig. 4). Flow cytometry on live splenic CD8^+^ T cells on days 25 and 40 postimmunization revealed naive and memory, effector, and central CD8^+^ T cell populations. In contrast, in the retina, >95% of cells were CD44^high^CD62L^low^. It should be noted that these cells lacked the markers CD25 and CD28 (data not shown). This indicates that, by the late phase of EAU, during CD8^+^ T cell accumulation, the majority of the CD8^+^ T cells have a phenotype consistent with Ag-activated T cells that have become T_EM_.

Further phenotyping using additional markers revealed that T_EM_ found in the retina have a different phenotype to their splenic counterparts (Fig. 4A). The expression of the surface marker Ly6C on T_EM_ in the spleen and the retina at day 25 EAU was similar; however, by day 40, Ly6C was significantly downregulated on retinal T_EM_ (Fig. 4B). CD69 expression is well known as an early activation marker on T cells, but it is also expressed on T_RM_ populations (31, 42). In the retina, CD69 was upregulated on T_EM_ compared with spleen at day 25 (Fig. 4A), and the percentage of cells expressing CD69 doubled by day 40 (Fig. 4B). Downregulation of Ly6C and increased expression of CD69 is a phenotype that has been reported in chronic LCMV infection, in which T_EM_ undergo changes in phenotype upon migration to infected tissue (23, 43). CD103 is a key marker found on T_RM_ subsets (24). Naive CD8^+^ T cells express intermediate amounts of CD103, and this expression is downregulated upon activation. In this study, splenic T_EM_ expressed minimal CD103 (Fig. 4A) compared with CD44^low^ cells (data not shown). Within the retina, upregulation of CD103 was limited; ∼25% of these cells expressed this surface marker (Fig. 4B).

Retinal T_EM_ retain the potential to kill, as shown by the expression, after stimulation with PMA/ionomycin, of granzyme B (Fig. 4C). However, they have low levels of CD107a expression, which declines as disease progresses, indicating that they have not recently degranulated (44). To assess function further, we also tested the cytokine production by these cells. Between days 25 and 40, there was a significant decrease in the percentage of cells capable of producing IFN-γ in response to PMA/ionomycin (*p* < 0.05) (Fig. 4C). There was also a trend toward reduced production of TNF-α that was not statistically significant. Therefore, there is no evidence that the selective accumulation of retinal T_EM_ is accompanied by an increase in effector function. Collectively, the phenotype indicates that the retinal CD8^+^ T_EM_ have experienced Ag stimulation in vivo, but within the retina effector function is held in check.

### Upregulation of PD-1 on retinal T_EM_ correlates with lack of effector function

**FIGURE 5.**
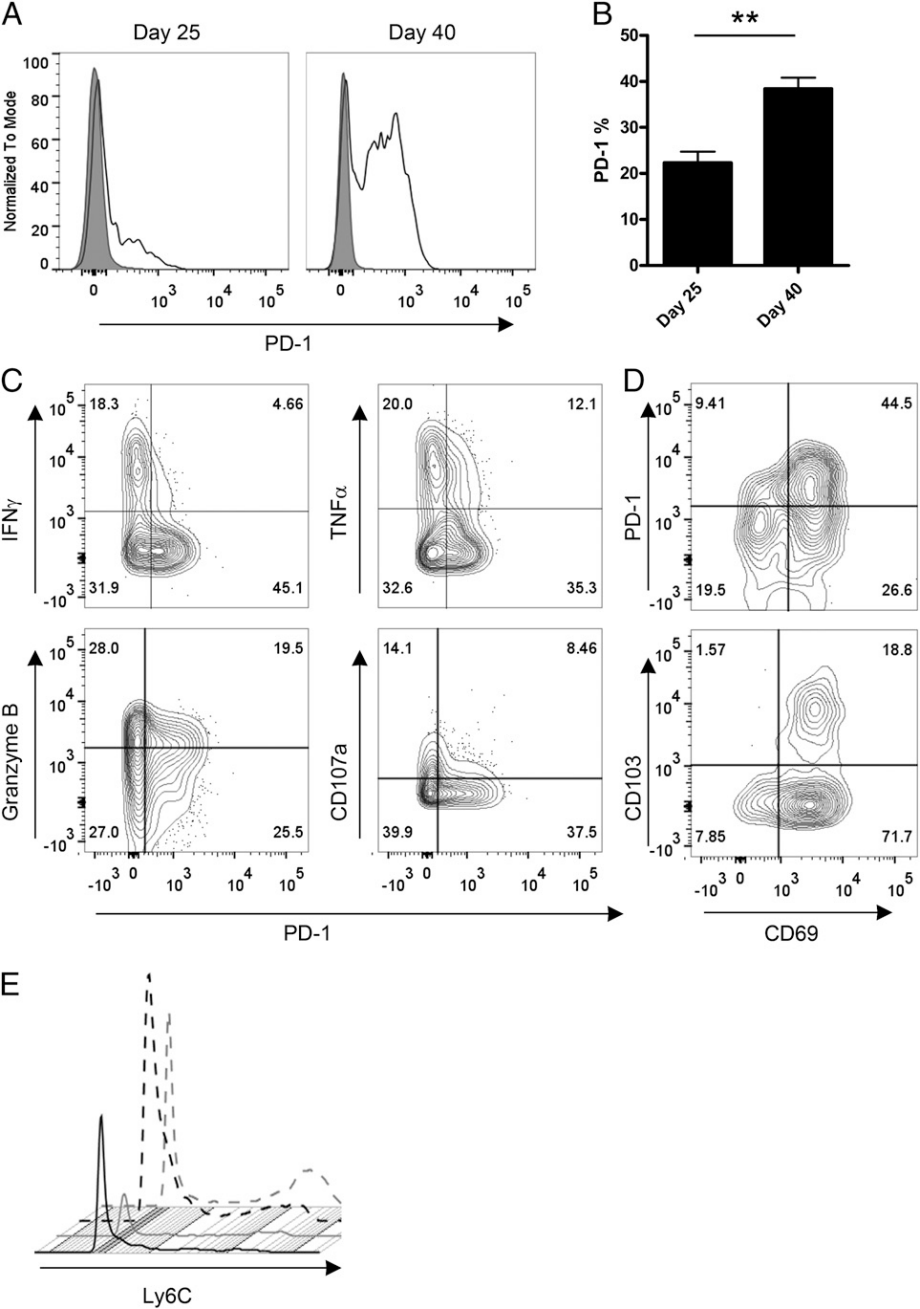
Upregulation of PD-1 correlates with a lack of effector function. CD8^+^ T cells isolated from the spleen or the retina from mice with EAU were examined by flow cytometry at 25 or 40 d postimmunization. After dissection, retinas were pooled together before analysis, at least three retinas per experiment. (**A**) Representative plots of the percentage of PD-1 expression on CD8^+^CD3^+^ cells in the spleen (gray filled histogram) and the retina (black histogram). (**B**) Combined retinal CD8^+^CD3^+^ T cell percentages for PD-1 expression. Data expressed as mean ± SEM. Data are representative of at least three independent experiments at each time point. (**C**–**E**) Retinal CD8^+^CD3^+^ T cells were examined on day 40 postimmunization for (C) cytokine and cytotoxic markers against PD-1 expression. (D) CD69 and PD-1 expression (*top panel*) and PD-1^+^ cells analyzed for CD69 and CD103 expression (*bottom panel*). (E) Ly6C expression from CD69^−^PD-1^−^ (dashed grey), CD69^+^PD-1^−^ (dashed black), CD69^−^PD-1^+^ (solid grey), and CD69^+^PD-1^+^ (solid black). Representative flow cytometry plots are shown. **p < 0.01.

If the T_EM_ that accumulate in the retina during the persistent phase of disease are responsive to local Ag, their lack of effector function must have some cause. There are well-known instances in which CD8^+^ Ag-specific T cells do accumulate in tissues without initiating their cytotoxic program, for example, in the presence of tumors (45). In our experiments, the T_EM_ in the retina could be chronically stimulated by autoantigens through their TCR. One outcome of chronic stimulation in CD8^+^ T cells is the development of an exhausted phenotype, which is described both in viral infections and tumor models (46). This is associated with a lack of effector function and upregulation of surface markers, including PD-1 receptor (47, 48). In T_EM_ recovered from the retinas of mice with EAU, we do find upregulation of PD-1, which significantly increased from days 25 to 40, consistent with continuous local activation (Fig. 5A, 5B) (*p* < 0.01). Dual staining of PD-1 with cytokines showed that PD-1 expression correlates with a lack of IFN-γ and TNF-α production along with CD107a and granzyme B, albeit to a lesser extent (Fig. 5C). Studying this PD-1^+^ population further, the majority of PD-1–positive cells were also CD69^+^ (5:1), whereas in the PD-1–negative compartment this ratio was lower (3:1). A small population of the PD-1^+^ CD69^+^ subset was also positive for CD103 (Fig. 5D). Ly6C, which aids cytolytic activity (49), segregated into the PD-1–negative population (Fig. 5E).

### FTY720 treatment during persistent EAU retains T_EM_ in tissue

**FIGURE 6.**
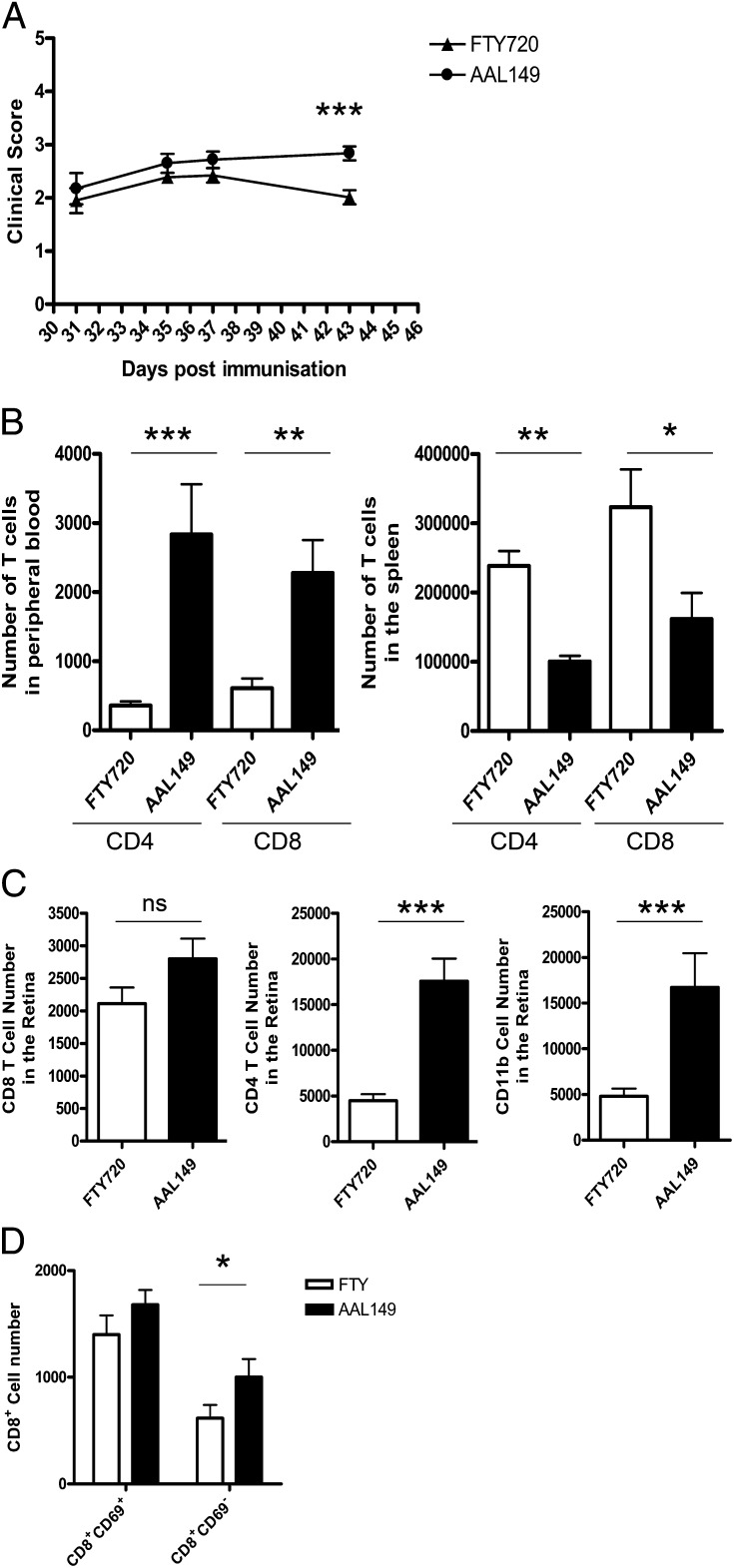
FTY720 treatment during chronic EAU reveals tissue residency of CD8^+^ T cells. Mice were immunized to induce EAU, and eyes were monitored by TEFI from day 30 onward. Groups of mice were treated with 0.3 mg/kg of either FTY720 or AAL149 (analog control) on day 35, and then every 24 h thereafter for 8 d. Peripheral blood, splenocytes, and retina were characterized and quantified 24 h after final treatment. (**A**) TEFI disease scores for each treatment group. (**B** and **C**) Average cell numbers for both treatment groups day 9 after treatment. (B) CD4^+^ and CD8^+^ T cells in peripheral blood (*left panel*) and the spleen (*right panel*). (C) CD8^+^, CD4^+^, and CD11b^+^ cells in the retina. (**D**) CD8^+^CD69^+^ and CD8^+^CD69^−^ cell numbers in the retina. Data represent two independent experiments. Each treatment has a minimum of 18 retinas. Data expressed as mean ± SEM. *p < 0.05, **p < 0.01, ***p < 0.001. ns, not significant.

The phenotype of the T_EM_ accumulated in the retina during persistent EAU has many similarities with populations of T_RM_ that arise following infection and localize in tissues such as the CNS, gut, lung, and the skin, where they are thought to provide local immune memory (24, 25, 27). These cells remain within their target tissues for extended periods of time without recirculating. To determine whether the T_EM_ that we have identified in persistent retinal inflammation are resident, we used FTY720 (Fingolimod) treatment, which arrests lymphocytes in the secondary lymphoid tissues and prevents trafficking back to tissues (50, 51). This leads to a decrease in lymphocytes within peripheral tissues and sites of inflammation, such as the brain and the retina, following FTY720 treatment (35, 52, 53). Mice with EAU were treated from day 35 postimmunization with 0.3 mg/kg FTY720 or control analog administered daily for 8 d before the CD8^+^ T cells in the retina were quantified. Treatment was associated with a significant decrease in EAU disease score (Fig. 6A) (*p* < 0.001). As expected, the numbers of CD8^+^ and CD4^+^ T cells were significantly reduced in the blood and significantly increased in the spleen, confirming that FTY720 interrupts the circulation of both CD4^+^ and CD8^+^ T cell populations (Fig. 6B). Analyses of the retinas of mice posttreatment with FTY720 (Fig. 6C) revealed a significant decrease in the number of CD4^+^ T cells and CD11b^+^ macrophages compared with the analog control AAL149 (*p* < 0.001). In contrast, no significant decrease was shown in the overall CD8^+^ T cell population, indicating that their *t*_1/2_ within the tissue was much greater than the CD4^+^ T cells. A subphenotype analysis of the CD8^+^ T cells remaining in the retina indicated that at least some of the CD8^+^CD69^−^ T cells were recirculating with kinetics similar to the CD4^+^ cells but that the CD69^+^CD8^+^ population was not (Fig. 6D).

### Retinal CD8^+^ T cells control local infiltrate

**FIGURE 7.**
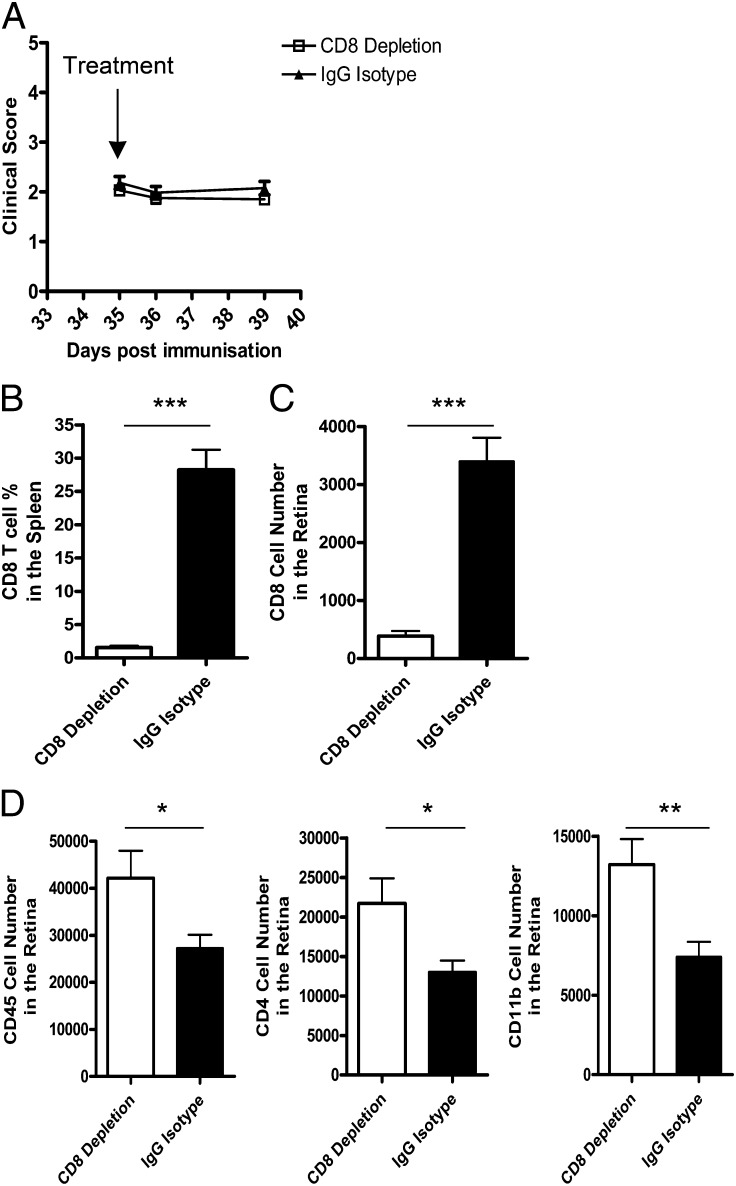
Retinal CD8^+^ T cells control local retinal infiltrate. Mice were immunized for EAU, and eyes were monitored by TEFI from day 30 onward. Groups of mice were treated with 250 μg of either CD8 depletion Abs or IgG isotype control on day 35 postimmunization for 4 d before quantification of cell populations by flow cytometry. (**A**) TEFI disease scores for each treatment group. (**B**) CD8^+^ T cell percentage in the spleen. (**C** and **D**) Average cell numbers in the retina for both treatment groups 4 d after treatment. (C) CD8^+^ T cells. (D) Total CD45^+^ cells, CD4^+^ T cells, and CD11b^+^ cells. Data represent four independent experiments; each treatment has a minimum of 16 retinas. Data expressed as mean ± SEM. *p < 0.05, **p < 0.01, ***p < 0.001.

FTY720 treatment reduces clinical disease, despite the persistence of CD8^+^ T cells within the retina; this is further evidence that these cells are not causing tissue damage. To determine other roles for these cells, we investigated whether the depletion of CD8^+^ T cells would affect the progression of persistent EAU and retinal cell infiltrate. Mice with EAU were given one treatment of either 250 μg CD8-depleting mAbs or an isotype control on day 35 postimmunization; 4 d later, retinal cell infiltrate was quantified by flow cytometry. As expected after treatment with depleting Abs, few CD8^+^ T cells were seen in the periphery and the retina (Fig. 7B, 7C). Although depletion of CD8^+^ T cells revealed little change in disease scores (Fig. 7A), a significant increase in total retinal infiltrate was observed (*p* < 0.05) (Fig. 7D). Treatment was associated with an increase in total CD45^+^ leukocytes, comprising CD4^+^ T cells and CD11b^+^ macrophages. We conclude that the removal of T_EM_ in the retina provokes an influx of pathogenic CD4^+^ T cells and CD11b^+^ cells, implying that exhausted tissue-resident T_EM_ play a role in limiting local disease.

## Discussion

In the current study, we report that CD8^+^ T cells that populate the inflamed retinal tissue late in the course of EAU are of T_EM_ phenotype, are Ag experienced, and exhibit features comparable with a chronically stimulated T_RM_ phenotype. These cells accumulate as disease progresses and have a long *t*_1/2_ in the tissue, as indicated by their retention following blockade of T cell circulation by FTY720. Depletion of this resident population from the retina leads to an influx of CD4^+^ T cells and CD11b^+^ macrophages. These data support the proposal that, during the primary peak of EAU, CD8^+^ T_EM_ migrate to the retina, where they encounter a specific microenvironment, which over time drives functional changes, including the upregulation of CD69 and PD-1, thereby allowing retention and accumulation of these Ag-specific cells in an exhausted state within the tissue, where they regulate local cell infiltrate.

We have established that there are changes in the levels of TCR and CD8 expression consistent with Ag-specific activation (40, 41). Testing Ag-specific activation confirms that a proportion of retinal resident CD8^+^ T cells responds to autoantigens, but they do not proliferate and they survive poorly when removed from the retina and cultured in IL-2 (data not shown). This is consistent with previous work demonstrating that T_RM_ do not survive well when removed from the brain (25). The selective accumulation of CD8^+^ T cells over time, despite similar levels of tissue inflammation and equivalent levels of CD4^+^ T cell infiltration, argues for a cognate recruitment process. Elegant studies in a diabetic NOD mouse model (54) demonstrate the absolute requirement of autoantigen recognition to the recruitment of CD8^+^ T cells to inflamed islet tissue. However, in other systems, only a very small number of autoantigen-specific cells is necessary for diabetes to develop (55). Taking the level of PD-1 activation as a sign of recent cognate interaction, we suggest that at least 40% of the T_EM_ are likely to be specific for autoantigen, rather than retained by bystander recruitment to the retina. However, without the development of Ag-specific class I tetramers and a complete understanding of epitope spreading, a precise accounting of cell numbers within the retina remains infeasible.

CD8^+^ T cell accumulation is a recognized feature of uveitis, but the role of these cells in the disease process is poorly defined (18). We now show that the CD8^+^ T cells that accumulate in the tissue during the persistent phase of disease (Fig. 1) are related to the T_RM_ cells that have been described in epithelial and neuronal tissues after viral infection (25, 32). We exploited previous findings that showed effective reduction in CD4^+^ T cells and CD11b^+^ macrophages in the retina within 24 h of treatment with FTY720 (35), revealing that most CD8^+^ T cells are retained. This is a novel observation that in a persistent autoimmune inflammatory setting CD8^+^ T cells have a prolonged tissue residency in contrast to their CD4^+^ T cell counterparts (Fig. 6). There is also a degree of heterogeneity in the CD8^+^ T cell populations within the retina; those that expressed CD69 were less affected by FTY720 treatment than CD69^−^CD8^+^ T cells. In the lymph node, upregulation of CD69 inhibits the exit of T cells mediated by the sphingosine 1-phosphate receptor 1 acting as a retention signal (56). Furthermore, expression of CD69 has a role in promoting the migration and retention of virus-infected CD8^+^ T cells to the lung, as cells lacking CD69 failed to traffic to the tissue efficiently (28). Therefore, the relative unresponsiveness of the CD69^+^CD8^+^ T cell population to FTY720 treatment in late EAU may implicate CD69 as the mediator of retinal T_EM_ persistence. Whether retinal T_EM_ are replenished from circulating memory cells over longer periods of time is yet to be explored.

Noteworthy differences were observed in the retinal T_EM_ compared with their peripheral counterparts (Fig. 4A, 4B), including the upregulation of CD69, which is consistent with reports following acute LCMV infection (23). CD103 expression on peripheral T_EM_ was minimal, but was expressed on ∼25% of the retinal T_EM_, but, unlike the increase established on CD8^+^ T_RM_ in other tissues (23), the expression was not upregulated in situ as disease progressed, rather it declined slightly. A similar pattern was seen for Ly6C with downregulation of this receptor segregating predominantly into the PD-1^+^ compartment. Moreover, an increase of PD-1 expression was seen over the course of disease and was associated with cells that lacked evidence of cytokine production and cytotoxic effector function (Fig. 5C).

T_RM_ can be identified by their expression of both CD69 and CD103 (24, 25, 27). CD103 interaction with its ligand, E-cadherin, has been shown to promote CD8^+^ T cell retention in the tissue (25, 28), and E-cadherin is present in the retina (57), but our data show that in persistent EAU CD103 is not required for CD8^+^ T cells to be retained, as both CD103^+^ and CD103^−^ cells remain in the retina after treatment with FTY720 (data not shown). However, it has been noted that persistent Ag stimulation prevents upregulation of CD103 expression in a chronic LCMV setting (24). Therefore, we conclude that chronic stimulation in the tissue leads to the difference in CD103 expression compared with T_RM_ initiated by acute viral infection.

In tissues such as the skin or gut, Ag is not required for the retention of T_RM_ and nonspecific inflammation is enough to produce these cells in other nonlymphoid tissues (24, 32). This implies that some tissue-specific T_RM_ are more influenced by their microenvironment than others; TGF-β can induce a T_RM_ phenotype in peripheral effector memory cells in ex vivo culture, and in vivo loss of TGF-β reduces the number of CD69^+^CD103^+^ cells in the small intestine (24). However, in studies of T_RM_ in tissues such as the brain, which is isolated by a blood barrier, local Ag is essential (24, 25). Hence, the differentiation or maintenance of CD8^+^ T_RM_ in peripheral tissue seems to be dependent on location (31). Whether our retinal T_EM_ require Ag to survive or are controlled by the microenvironment was not addressed directly in this study.

PD-1 is widely used as a marker of T cell exhaustion and its blockade has been successful in experimental and clinical therapy for tumors (58, 59). T cell exhaustion due to chronic viral infections or tumor immunology leads to a hierarchical loss of function in effector CD8^+^ T cells (46, 60). Reversal of this exhausted phenotype, by blocking PD-1 interactions, results in an increase in viral clearance and reduced tumor growth (48, 61–63). Some exhausted T cells also coexpress other inhibitory molecules such as TIM-3 and LAG-3, and blockade of these inhibitory receptors in unison with PD-1 can enhance the restoration of functionality (61, 64). Furthermore, it has been shown that complex coexpression of these inhibitory receptors exists depending on the severity of viral infection (61). We found negligible expression of TIM-3 and LAG-3 on the T_EM_ in the retina (data not shown), which indicates an intermediate level of exhaustion in this model.

Expression of PD-1 is found constitutively in retinal neurons, and with active retinal inflammation the ligand programmed cell death ligand 1 (PD-L1) is upregulated (65, 66). Similarly, PD-L1 levels on astrocytes and microglia in the brain are elevated during disease (67). Interestingly, under inflammatory conditions, retinal pigment epithelium expresses PD-L1 and suppresses T cell activation in vitro (68).

Finally, there is the question of whether exhausted T_EM_ tissue-resident cells have a functional role. Depleting these cells leads to a significant increase in the number of retinal CD4^+^ T cells and macrophages, and the number of cells recruited is much greater than the number of CD8^+^ cells that are lost. This suggests that CD8^+^ T cells may play a part in regulating the level of cellular infiltrate. This might be through engagement of PD-1 by other cells locally. PD-1 signaling can limit IFN-γ production and proliferation (69), as well as control regulatory T cells (66). Alternatively, other less well-defined mechanisms may be involved. Taken together, the data indicate that retinal resident T_EM_ are a potential source of local regulation during persistent intraocular inflammation.
